# Evaluating microbial contaminations of alternative heating oils

**DOI:** 10.1002/elsc.202300010

**Published:** 2023-05-05

**Authors:** Maximilian J. Surger, Katharina Mayer, Karthik Shivaram, Felix Stibany, Wilfried Plum, Andreas Schäffer, Simon Eiden, Lars M. Blank

**Affiliations:** ^1^ Institute of Applied Microbiology (iAMB) Aachen Biology and Biotechnology (ABBt) RWTH Aachen University Aachen Germany; ^2^ Institute for Environmental Research RWTH Aachen University Aachen Germany; ^3^ OWI Science for Fuels gGmbH Herzogenrath Germany; ^4^ TEC4FUELS GmbH Herzogenrath Germany

**Keywords:** CO_2_ monitoring, heating oil storage, microbial activity, oxymethylene ethers, paraffinic heating oils

## Abstract

Since 2008, European and German legislative initiatives for climate protection and reduced dependency on fossil resources led to the introduction of biofuels as CO_2_‐reduced alternatives in the heating oil sector. In the case of biodiesel, customers were confronted with accelerated microbial contaminations during storage. Since then, other fuel alternatives, like hydrogenated vegetable oils (HVOs), gas‐to‐liquid (GtL) products, or oxymethylene ether (OME) have been developed. In this study, we use online monitoring of microbial CO_2_ production and the simulation of onset of microbial contamination to investigate the contamination potential of fuel alternatives during storage. As references, fossil heating oil of German refineries are used. Biodiesel blends with fossil heating oils confirmed the promotion of microbial activity. In stark contrast, OMEs have an antimicrobial effect. The paraffinic Fischer–Tropsch products and biogenic hydrogenation products demonstrate to be at least as resistant to microbial contamination as fossil heating oils despite allowing a diversity of representative microbes. Through mass spectrometry, elemental analysis, and microbial sequencing, we can discuss fuel properties that affect microbial contaminations. In summary, novel, non‐fossil heating oils show clear differences in microbial resistance during long‐term storage. Designing blends with an intrinsic resistance against microbial contamination and hence reduced activity might be an option.

**Abbreviations**
EL: extra light; HEL, heating oil extra light; UCO, used cooking oil; OFS, surface tension; MO, microorganisms

## 1 INTRODUCTION

### 1.1 Microbial contamination during heating oil storage

Ten million households and 29 million people in Germany, approximately 25% of the population, are stably supplied with heat via heating oil extra light (EL). The industry expects that in the course of the Climate Protection Plan 2050, fossil fuels will be successively replaced by more climate‐friendly alternatives, but that the importance of liquid fuels will be maintained due to the existing infrastructure, their energy density, and storage stability (https://www.bmu.de/publikation/klimaschutzplan‐2050‐klimaschutzpolitische‐grundsaetze‐und‐ziele‐der‐bundesregierung, accessed 8 December 2021; Mineral oil industry association e.V. Annual Report: Liquid fuels move Germany – also 2050, https://en2x.de/service/publikationen/, accessed 8 December 2021; Mineral oil industry association e.V. Annual Report: Electricity in the tank – New energy in the pipeline, https://en2x.de/service/publikationen/, accessed 8 December 2021; Mineral oil industry association e.V. Annual Report: Clean fuels for all, https://en2x.de/service/publikationen/, accessed 8 December 2021) [1]. However, this storage stability is increasingly discussed critically by consumers and industry. In addition to abiotic aging, the focus is primarily on the susceptibility to microbial contamination and accompanying damage (i.e., biofouling) of burner systems [2]. Due to a continuous increase in storage time in private households occurrence and intensity of microbial contamination of heating oil EL will increase and the challenge will likely continue to grow in importance for the industry [3].

**PRACTICAL APPLICATION**

The online monitoring of microbially produced CO_2_ as a measure for microbial activity allows the evaluation of microbial contaminations within two‐phase culture systems, such as the storage of heating oil products. The market for heating oil products is undergoing rapid changes in its composition away from fossil resources to CO_2_‐neutral alternatives. The first alternative fuels represent an increased risk to burner systems due to their microbial susceptibility. The application of the two‐phase culture system, a representative microbial inoculum, and CO_2_ online monitoring to current fuel developments, presented in this study, make this risk assessable. This study provides the basis for the further development of a rapid test format that is expected to support the development of microbial‐resistant blending strategies.

Fundamental to the outbreak of microbial contamination in heating oil storage is the presence of water, mainly introduced by atmospheric moisture via tank ventilation, in form of a separate free water phase or emulsions. Volumes of 1–3 μL and water activity (*a*_w_) of >0.8 are the basis of microbial contamination [4, 5, 6, 7, 8, 9, 10, 11, 12, 13]. Further progression of microbial contamination is significantly influenced by the availability of nitrogen. Nitrogen sources in mineral oil products are ammonium salts and heterocycles such as anilines, pyridines, quinolines (alkaline) or indoles, and carbazoles (non‐alkaline) [14, 15, 16]. Other factors that drive microbial contamination are osmotic pressure, salinity, oxygen availability, carbon sources (hydrocarbons), temperature, and Ph [13].

### 1.2 Use of alternative fuels

Since 2008, a large number of political campaigns and legislative initiatives (for instance EEWärmeG, October 2015 [Germany]; REDII, 2018/2001; Climate Protection Plan 2050 [Germany]; Fuel Emissions Trading Act [BEHG]) have called for an increasing fixed share of renewable energies, also in the heating market (Mineral oil industry association e.V. Annual Report: Clean fuels for all, https://en2x.de/service/publikationen/, accessed 8 December 2021) [17, 18]. To continue to use the existing predominantly private infrastructure for liquid fuels and to store energy generated from solar, wind, and hydropower (“power‐to‐liquid (PtL)”), liquid fuels continue to exist [19] (https://www.bmu.de/publikation/klimaschutzplan‐2050‐klimaschutzpolitische‐grundsaetze‐und‐ziele‐der‐bundesregierung, accessed 14 December 2021). In this context, fossil fuels are being potentially replaced by new alternative CO_2_‐reduced or ‐neutral biogenic or synthetic fuels, which can be distinguished based on feedstock, manufacturing pathway, and possibly microbial susceptibility (Mineral oil industry association e.V. Annual Report: Clean fuels for all, https://en2x.de/service/publikationen/, accessed 8 December 2021). The environment of heating oil storage, in which microbial contaminations develop is thus subject to massive changes concerning substrate availability or diversity, water solubility, and toxicity.

### 1.3 Relevance of biodiesel for microbiology

Biodiesel or fatty acid methyl esters (FAMEs) are produced via transesterification of vegetable oil‐based triglycerides and methanol. By‐products are phospholipids and free fatty acids. Antioxidants (in ppm) are added and plant fertilizers are present in traces (https://biokraftstoffe.fnr.de/kraftstoffe/biodiesel/, accessed 15 December 2021). In Germany, the most important feedstock in 2021 was recycled used cooking oil (37%, Used Cooking Oil, UCO), followed by rapeseed oil (33%), palm oil (25%), and soybean oil and sunflower oil (1% and 3%, respectively) (https://www.ufop.de/files/8616/3420/4006/Web_RZ_UFOP_1801_Biodieselauszug_2021_141021_ALO.pdf, accessed 18 June 2022). Celebrated for its environmental friendliness due to generally low emissions, especially CO_2_ neutrality and biodegradability, FAME soon revealed limited long‐term storage stability in heating oil applications due to low autooxidation resistance and biodegradability or microbial susceptibility, resulting in biofouling [20, 21]. In Germany, a realistic future market target is the blending of up to 20% biodiesel to heating oil EL.

### 1.4 Microbial relevance of paraffinic fuel alternatives

The paraffinic fuel alternatives currently include hydrogenated vegetable oils (HVOs, biogenic) and the products of Fischer–Tropsch synthesis (XtL, synthetic) [19]. HVOs can be derived from the same feedstocks as biodiesel (FAME) (https://www.ble.de/SharedDocs/Downloads/DE/Klima‐Energie/Nachhaltige‐Biomasseherstellung/Evaluationsbericht_2015.html, accessed 15 December 2021). HVOs are produced in two steps. Hydrogenation saturates double bonds and removes oxygen from ester bonds by splitting off water or CO_2_ (https://patents.google.com/patent/EP1741767B1/en, accessed 19 December 2021). Unbranched alkanes with 15–18 carbon atoms are formed, corresponding to plant fatty acids. In a second step, most of the alkanes are converted by isomerization into iso‐alkanes with advantageous physical properties (https://patents.google.com/patent/EP1741767B1/en, accessed 19 December 2021; https://biokraftstoffe.fnr.de/kraftstoffe/hydrierte‐pflanzenoele‐hvo, accessed 20 December 2021; https://patents.google.com/patent/US20040230085A1/en, accessed 20 December 2021; https://docplayer.org/76103112‐Herstellung‐thg‐reduzierter‐fluessiger‐kraft‐und‐brennstoffe‐herstellung‐thg‐reduzierter‐fluessiger‐kraft‐und‐brennstoffe‐kurzstudie.html, accessed 20 December 2021) [19, 22–24]. The starting material of the Fischer–Tropsch process is synthesis gas consisting of hydrogen and carbon monoxide. The products of the Fischer–Tropsch synthesis are very long‐chain unbranched aliphatics [25]. Hydro‐cracking and hydro‐isomerization also yield predominantly branched alkanes with chain lengths in the middle distillate range (https://www.ble.de/SharedDocs/Downloads/DE/Klima‐Energie/Nachhaltige‐Biomasseherstellung/Evaluationsbericht_2015.html, accessed 15 December 2021; https://docplayer.org/76103112‐Herstellung‐thg‐reduzierter‐fluessiger‐kraft‐und‐brennstoffe‐herstellung‐thg‐reduzierter‐fluessiger‐kraft‐und‐brennstoffe‐kurzstudie.html, accessed 20 December 2021; https://www.dena.de/newsroom/publikationsdetailansicht/pub/studie‐biomass‐to‐liquid‐btl‐realisierungsstudie/, accessed 20 December 2021) [19, 26–28]. The reality for the use of paraffinic fuel alternatives in the heating oil sector (heating oil EL P) is a draft technical specification DIN/TS 51603‐8, which in principle allows replacing fossil heating oil EL to 100% by paraffins. The technical limitation is the reduced lubricity and density due to the lack of aromatics. This could be compensated by blending with biodiesel, which is currently not permitted. A market‐realistic target is the blending of 50% paraffinic fuel alternatives to fossil EL heating oil or 20% biodiesel and 50% paraffinic fuel alternatives to fossil EL heating oil (https://docplayer.org/76103112‐Herstellung‐thg‐reduzierter‐fluessiger‐kraft‐und‐brennstoffe‐herstellung‐thg‐reduzierter‐fluessiger‐kraft‐und‐brennstoffe‐kurzstudie.html, accessed 20 December 2021) [29, 30, 31]. Practical empirical data and scientific studies on biodegradation performance, biomass development, microbial diversity, and effects of contamination are currently not documented.

### 1.5 Microbial relevance of oxymethylene ethers

The starting product of oxymethylene ether (OME) synthesis is methanol, which is produced from synthesis gas. OME is produced via formaldehyde or trioxane via polymerization reactions with further methanol and formaldehyde. The OMEs with the chemical structure H_3_C–O–(CH_2_O)*_n_*–CH_3_ (OME*_n_*) contain no C–C bonds, carbon atoms are connected via ether bridges. For application as an alternative fuel in the diesel/heating oil sector, a blend of the oligomers OME_3‐5_ is used and an admixture of up to 15% v/v is targeted (https://www.ingenieur.de/technik/forschung/diesel‐ersatzkraftstoff‐ome‐fuer‐klimaneutralitaet‐sorgen/, accessed 20 December 2021; https://www.ffe.de/veroeffentlichungen/welche‐strombasierten‐kraftstoffe‐sind‐im‐zukuenftigen‐energiesystem‐relevant/, accessed 20 December 2021) [32, 33, 34, 35]. Practical experience and scientific studies on biomass development, microbial diversity, and effects of contamination are not documented at present.

### 1.6 Objective of this study

We investigated microbial susceptibility of the latest alternative heating oils and their blends during storage. We used continuous online monitoring of microbial CO_2_ production as a measure of microbial activity (catabolic activity and biomass development) [36]. A defined microbial mixed culture was used for a simulated onset of microbial contamination [37]. As a reference, the promotion of microbial activity by biodiesel could be confirmed. For the first time, the resistance of OMEs and paraffinic alternatives to microbial contamination is reported and quantified. In addition, crucial properties of alternative fuels for promoting or inhibiting microbial contamination are investigated and discussed.

## 2 MATERIALS AND METHODS

### 2.1 Microbial strains and storage cultures

**FIGURE 1:**
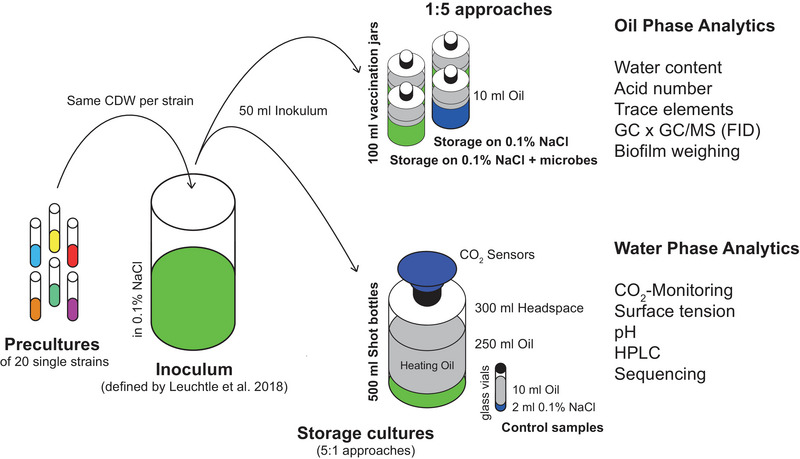
Overview of used storage cultures. Shown are the two different types of storage cultures, used in this study, and the setup of those storage cultures using a defined inoculum of 20 strains (Table 1), representative of heating oil contamination, as defined by Leuchtle et al. (2018) [37]. The classical storage cultures (5:1 approaches) are intended for the CO_2_ measurement and water phase analytics (surface tension, pH, HPLC, sequencing), and the alternative storage cultures (1:5 approaches) are meant for oil phase analytics (water content, acid number, trace elements, GC × GC/MS or GC × GC/FID) and biofilm quantification. FID, flame ionization detector; GC, gas‐chromatography; HPLC, high‐performance liquid chromatography; MS, mass spectrometry.

To mimic an oil‐storage‐tank (1:5 approaches) 50 mL (100 mL in case of OME blend investigation) of the free water phase, consisting of 0.1% NaCl, was overlaid with about 250 mL (500 mL in case of OME blend investigation) fossil heating oil or alternative fuel within a 500 mL shot bottle (1 L shot bottle in case of OME blend investigation), resulting in a 300 mL (550 mL in case of OME blend investigation) headspace. To investigate the effect of microbes on fuels 50 mL of free water phase were overlaid with 10 mL fossil heating oil or alternative fuel within a 100 mL glass bottle with constant air supply via needle and sterile filter (5:1 approaches) (Figure 1). A volume of 800 mL water phase was inoculated with a mixture of 20 representative heating oil microbes as defined by Leuchtle et al. [37] including an amount per strain corresponding to 16 mg CDW (a total of 320 mg CDW for all microbes). The microbial mixture did not contain anaerobes as oil storage tanks are ventilated [37]. The microbes used are found in Table 1. The precultures were made in LB (10 g/L peptone, 10 g/L yeast extract, 5 g/L NaCl medium), YEP medium (20 g/L peptone, 20 g/L glucose, 10 g/L yeast extract), YEPS medium (10 g/L peptone, 10 g/L yeast extract, 10 g/L sucrose), potato extract glucose bouillon (PEGB, 26,5 g/L), or ME medium (20 g/L malt extract, 1 g/L peptone) for bacteria, yeasts, *Ustilago maydis*, *Rhodotorula mucilaginosa*, and molds, respectively. Precultures of single strains consisted of 100 mL medium in 1 L Erlenmeyer flasks without baffles grown into early stationary phase. Those single cultures were shaken at 200 rpm and incubated at 30°C. The precultures were washed with 0.1% NaCl before use. The shot bottles were not shaken, were kept in the dark, and at room temperature.

**TABLE 1:** Strains used in this study and for the defined inoculum of the heating oil tank simulation [37].

Strain	Database number	Source
*Acinetobacter beijernickii*	DSM 22901	DSMZ
*Acinetobacter venetianus*	DSM 23050	DSMZ
*Burkholderia cepacia*	DSM 7288	DSMZ
*Burkholderia xenovorans*	–	Leuchtle et al. (2018)
*Micrococcus luteus*	–	Leuchtle et al. (2018)
*Micrococcus yunnanensis*	DSM 21948	DSMZ
*Pseudomonas fluorescens*	–	Leuchtle et al. (2018)
*Pseudomonas poae*	–	Leuchtle et al. (2018)
*Candida cylindracea*	DSM 2031	DSMZ
*Debaryomyces hansenii*	DSM 70244	DSMZ
*Debaryomyces polymorphus*	DSM 70816	DSMZ
*Pichia membranifaciens*	DSM 21959	DSMZ
*Raffaelea* sp.	–	Leuchtle et al. (2018)
*Rhodotorula mucilaginosa*	DSM 18184	DSMZ
*Ustilago maydis*	–	Leuchtle et al. (2018)
*Yarrowia deformans*	CBS 2071	CBS‐KNAW
*Yarrowia lipolytica*	–	Leuchtle et al. (2018)
*Paecilomyces lilacinus*	DSM 846	DSMZ
*Penicillium chrysogenum*	DSM 21171	DSMZ
*Penicillium citrinum*	–	Leuchtle et al. (2018)

### 2.2 CO_2_ measurement

For the measurement of CO_2_ development, we used the BCP‐CO_2_ system (BlueSens Gas Sensor GmbH). The CO_2_ concentration is monitored by a source of infra‐red light, which is weakened by the analyte gas and reflected into the detector unit of the sensor. The sensor was attached airtight to the opening of a culture vessel, which was in this study a 1 L shot bottle. Measurements were performed without air exchange up to 2 weeks. Oxygen availability of microbial metabolism limits longer runtimes [36].

### 2.3 Surface tension and pH measurements

The Force Tensiometer K11 (KRÜSS) and a pH meter (Hanna Instruments) were used to measure the surface tension and pH of the separate water phases in the storage cultures. Five‐hundred 500microliter portions of sterile filtered water phase were used.

### 2.4 High‐performance liquid chromatography (HPLC) measurements

Samples of the free water phase were harvested after 1 week and analyzed by HPLC without further treatment. The control samples consisted of 2 mL 0.1% NaCl without microbes under 10 mL of fuel in 12 mL glass vials. An UltiMate 3000 series instrument from Dionex‐Themo Scientific was used for HPLC‐RI analysis. A Metab‐AAC HPLC column of the BF series from ISERA (300 × 8 mm), intended for the analysis of sugars, organic acids, and alcohols, was used. 5 mM H_2_SO_4_ was applied as running buffer at a flow rate of 0.6 mL/min. The column oven was kept at 75°C. The alcohols were detected via the IR detector unit Refracto Max 521 (running at 35°C with a data collection rate of 10 Hz and an integrator range of 500 μRIU/V). Quantification was based on peak areas and occurred via an external standard curve of the compound.

### 2.5 Gas‐chromatography (GC‐FID) for oxymethylene ether (OME) solutions

A Trace‐5 gas chromatograph from Thermo Fisher with a VF‐WAXms column (60 m/0.25 mm/0.25 μm) from Agilent was used for GC analysis. Helium was applied as carrier gas at a pressure of 1.3 bar. The injector and FID detector were operated at 250°C. The following temperature program was run in the column oven: 10 min at 50°C, with 8°C/min to 250°C, 30 min at 250°C. The mass recovery was calculated as the difference between the mass of the total sample weighed in and the mass of the OME components found and quantified in the sample by GC‐FID. Quantification is performed by peak area comparison with the 1‐pentanol standard used and taking into account pre‐calculated correction factors for the individual OME components. The GC method does not take into account the OME component OME‐6.

### 2.6 Determination of the water content in oil phases

The water content was determined in accordance with DIN EN ISO 12937 via coulometric titration as per Karl Fischer for mineral oil products with boiling points below 390°C [38].

### 2.7 Determination of the acid number in oil phases

The acid number was determined according to EN 14104 “Products of vegetable and animal fats and oils – Fatty acid methyl esters (FAMEs) – Determination of acid number” [39].

### 2.8 Trace element analysis in oil phases

A screening of trace elements in oil phases was carried out as a contract analysis at ASG Analytik‐Service AG using mass spectrometry with inductively coupled plasma (ICP‐MS).

### 2.9 Determination of the nitrogen content in oil phases

The determination of the nitrogen content in oil phases was carried out as a contract analysis at ASG Analytik‐Service AG using the combustion method with a chemiluminescence detector according to DIN 51444 [40].

### 2.10 Extraction of genomic DNA from storage cultures

For the isolation of genomic DNA from water phases of storage cultures or the inoculum used for storage cultures, a mechanical extraction procedure in combination with chemical purification was chosen to achieve minimal discrimination of individual bacterial, yeast, or mold strains. When extracting from water phases of completed storage cultures, always the complete water phase was collected and extracted as one sample, that is, including biofilm of the interface and the sediment.

The cell suspension was centrifuged in a 50 mL reaction tube (10 min, 12,000 rpm, 4°C). The supernatant was removed by suction. The remaining supernatant including the cell pellet was frozen overnight at −20°C. The frozen remaining supernatant was removed using a SCANVAC CoolSafe bench top freeze dryer (Labogene) overnight and the cell pellet was completely dried. The cell pellet was resuspended in 1.2 mL of lysis buffer (2% Triton X‐100, 1% SDS, 100 mM NaCl, 1 mM EDTA, 10 mM Tris‐HCl pH 8.2). The cell suspension was divided into two 2 mL screw cap reaction tubes (600 μL each) (MP Biomedicals) filled with 0.7 g ceramic beads (soilGEN, diameter 125–250 μm). For mechanical disruption, the FastPrep 24 5G homogenizer (MP Biomedicals) was used with the following program: 4 m/s, three cycles of 30 s each (stored on ice for 30 s in between). The reaction tubes were then stored on ice and centrifuged at 14,000 rpm, 4°C until the foam settled completely. The supernatant (approximately 600 μL) was transferred to new 2 mL reaction tubes. One‐hundred microliter NaCl (5 M) and 80 μL CTAB (10% w/v in 0.7 M NaCl) heated to 37°C were added to each tube and mixed thoroughly. The suspension was incubated at 65°C, 500 rpm for 10 min. Six‐hundred microliter of phenol:chloroform:isoamyl alcohol (25:24:1) was added. The mixture was shaken for at least 30 s and then centrifuged at 12,000 rpm, 25°C for 5 min. The upper phase was transferred to a new 2 mL reaction tube. Six‐hundred microliter of chloroform:isoamyl alcohol (49:1) was added and shaken thoroughly. The suspension was centrifuged at 12,000 rpm, 25°C for 5 min and the upper phase was transferred to a new 1.5 mL reaction tube. Five‐hundred microliter of isopropanol (ice cold) was added and DNA was precipitated by inverting 30 times. After centrifugation for 10 min at 12,000 rpm, 25°C the supernatant was removed and 700 μL of 70% v/v ethanol (cold) was added to the pellet. The mixture was incubated at 25°C for 15 min and then centrifuged at12,000 rpm, 25°C for 5 min. The supernatant was removed as far as possible and the DNA pellet was air dried. The pellet was resuspended in 25 μL Tris‐HCl (10 mM, pH 8.0) overnight at 37°C, 500 rpm. The mixtures of one culture sample, split before cell disruption, were recombined and stored at 4°C.

### 2.11 Sequencing and bioinformatics analysis

Libraries for Illumina sequencing were prepared with the Illumina DNA Prep (M) Tagmentation kit, using 100 ng of DNA as input. Sequencing was performed in a 2 × 151 bp mode on a NextSeq 550 using a 300 cycles reagent with unique dual indexing (IDT for Illumina DNA/RNA UD Indexes, Tagmentation). Library preparation and sequencing were done by the Institute of Medical Microbiology and Hygiene, NCCT Microbiology (Tübingen, DE).

The raw Illumina data conversion and demultiplexing were performed with the nxf‐bcl pipeline [41]. The raw reads were first filtered and trimmed using the nxf‐fastqc pipeline [42], which uses the fastp program with default filter and trimming parameters. The filtered reads were then mapped with minimap2 [42] against a custom database that contains the genomes from the microbial mix used as inoculum. The alignment PAF files were filtered to retain only full‐high‐quality alignments (>120 residues matching per read and mapping quality ≥50). The alignment files were used to extract summary information about the number of reads mapping to each species. The data processing was done by Dr. rer. nat. Angel Angelov (Freising, DE).

From reads per species per sample the shares of species per sample were calculated. The fold change of the shares was calculated between samples taken at the end of the storage cultures and samples of the inoculum used to start the storage cultures.

### 2.12 Identification/semi‐quantification by GC x GC/MS and quantification by GCxGC/FID of oil phases

Before injection 25–30 mg of the oil phase was dissolved in 20 mL of *n*‐pentane and 10 μL of cholestane (2 g/L) or octanoic acid ethyl ester (C8:0‐Et, 2 g/L) was added as internal standard.

Analysis was done using a GC x GC system (Thermo Scientific, JEOL, and Zoex) of Brechbühler AG (Schlieren, Switzerland) [43]. One microliter sample was injected using a PTV on‐column injector with an untreated fused silica precolumn (0.5 m × 0.53 mm ID). Helium was applied as carrier gas with 1 mL/min. The compounds were eluted on a polar DB‐17 HT column (15 m × 0.25 mm ID, 0.15 μm film) in the first dimension and on a non‐polar PS‐255 column (3 m × 0.15 mm ID, 0.04 μm film) in the second dimension. Per column, the following program was used: 3 min at 36°C, 5°C/min to 320°C. Before the second column, the compounds were trapped in a loop by a cold jet and released after a modulation time of 7.0–7.5 hot jet hot‐jet. The second column was followed by a time of flight mass spectrometer (ToF‐MS) for identification and semi‐quantification with a separation range of 40–800 amu and working at 50 Hz. Alternatively, the second column was connected to a flame ionization detector (FID) working at 350°C with 350 mL/min air, 35 mL/min H_2_, and 30 mL/min N_2_. The measurement was done by Laboratory Lommatzsch & Säger (Cologne, Germany).

### 2.13 Biofilm weighing

Biofilm weight was calculated by subtracting the weight of empty filters, which were incubated overnight at 100°C, from the weight of filters loaded with a harvested biofilm that were also incubated overnight at 100°C. Biofilm was harvested from four biological replicates of 5:1 approaches per oil phase using an inoculation loop. Glass fiber filters with 0.4 μm diameter were used (GF‐5, Macherey Nagel). Weight was measured using a moisture analyzer (MAC 50/1/NH, RADWAG).

### 2.14 Extra light heating oils used in this study

The fossil heating oils (HEL2 and HEL3) used in this study represent individual batches from different German refineries, with different crude oil sources and production conditions.

### 2.15 Alternative fuels used in this study

The alternative fuels used in this study are: Rapeseed oil methyl ester, used cooking oil methyl ester, gas‐to‐liquids (GtL), HVOs, and OMEs. They represent individual batches from different German production sites of international companies of the mineral oil or food industry.

## 3 RESULTS

Two complementary microbial culture approaches were used in this work. In the storage cultures with 250 mL of oil phase and 50 mL of water phase, therefore also called 5:1 cultures, the effect of the oil phase on the contaminating microbes was investigated. The analyses used were the measurement of microbially produced CO_2_ as a measure of microbial activity (catabolic activity of the biomass present) [36] and water phase analysis. Water phase analysis included surface tension and pH analysis as indicators of the presence of oil phase extracts or microbial metabolites, HPLC‐UV‐RI measurement to accurately identify and quantify these substances, and sequencing of extracted gDNA to estimate microbial diversity. These cultures were performed under non‐permanent aeration and were limited in time to maximal 3 weeks, depending on the available oxygen (Figure 1) [36].

In the alternative storage cultures with 10 mL oil phase and 50 mL water phase, therefore also called 1:5 cultures, the effect of the contaminating microbes on the oil phase was investigated and microbially relevant parameters of the oil phases were determined. These cultures were not subject to oxygen limitation and hence, run longer. Oil phase analysis included water content, acid number, trace elements (plus nitrogen), GC‐FID, and GC x GC‐MS (Figure 1).

### 3.1 Toxic degradation products of OME blends prevent microbial activity

**FIGURE 2:**
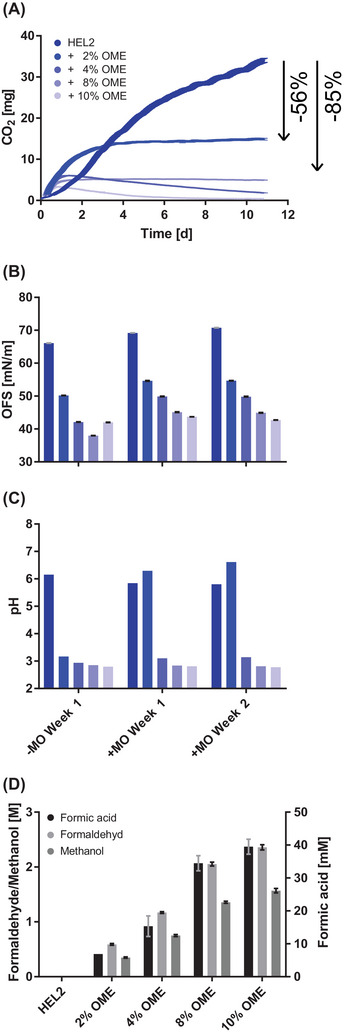
Storage culture of OME admixtures in fossil heating oil. Microbial activity in 11‐day storage of heating oil HEL2 and admixtures of 2%–10% OME measured by CO_2_ accumulation in the headspace of the culture bottle is shown. Plotted is the continuous CO_2_ measurement of individual biological replicates (A). The surface tension of free water phases under the tested oil phases after 1 and 2 weeks with and without microbes is shown. Plotted are five technical replicates each (B). The pH of the free water phases after 1 and 2 weeks with and without microbes is shown. Plotted are individual biological replicates (C). Methanol, formaldehyde, and formic acid concentrations as a result of HPLC analysis of the free water phases after 1 week without microbes are shown. Two technical replicates each are plotted (D). HPLC, high‐performance liquid chromatography; OME, oxymethylene ether.

During a 5:1 culture of 2 weeks, the addition of 2% OME to HEL2 led to a reduction of the microbial activity, measured by CO_2_ accumulation. In 550 mL headspace, 34 mg CO_2_ accumulated in the case of pure fossil heating oil, but only 15 mg CO_2_ in the case of the addition of 2% OME, which corresponds to a reduction by 56%. A maximum suppression of microbial activity by 85% was already observed for an admixture of 4% OME (Figure 2A).

The measurement of pH and surface tension of the free water phase indicated a decreasing surface tension and pH value of the non‐buffered water phase with increasing OME content of the oil phase in the presence and absence of microbes. In the presence of microbes, the pH remains neutral at an admixture of 2% OME (Figure 2B,C). In control samples without microbes, formaldehyde was detected by HPLC‐UV‐RI at concentrations of up to 2.4 M, methanol at concentrations up to 1.6 M, and formic acid at concentrations up to 40 mM after 1 week, depending on OME admixture (Figure 2D).

The integrity of the OME solution used for blending (0.2% OME‐2; 46.97% OME‐3; 29.40% OME‐4; 16.66% OME‐5; 5.57% OME‐6) was confirmed by GC analysis (Figure S1).

**FIGURE 3:**
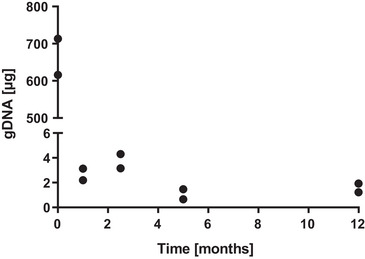
Biomass development in long‐term storage of 10% oxymethylene ether (OME) blends in fossil heating oil HEL2. As an indicator of biomass, the amount of genomic DNA extractable from whole cells is plotted from each of two biological replicates.

To test the sustainability of the antimicrobial effect of the OME admixture, long‐term storage of the blend of HEL2 and 10% OME in closed shot bottles was carried out for 1 year (5:1 culture). After one, two and a half, five, and twelve months, the biomass formed was estimated via the quantification of genomic DNA from intact cells. Based on the extractable DNA concentration, a reduction in intact biomass to below 1% occurred within the first month and no re‐increase was observed within 12 months (Figure 3).

### 3.2 Limited microbial activity below gas‐to‐liquid and hydrogenated vegetable oils

**FIGURE 4:**
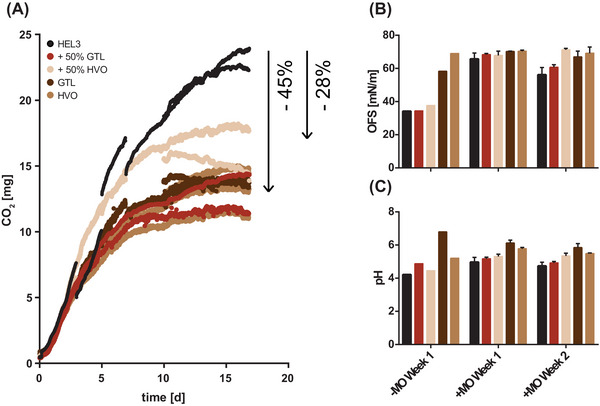
Storage cultures of gas‐to‐liquids (GtL) and hydrogenated vegetable oil (HVO) blends. Microbial activity in a 17‐day storage culture with heating oil HEL3, fossil heating oil with 50% admixture of GtL or HVO, and pure GtL or HVO is shown. Microbial activity is measured by the sum of CO_2_ accumulation in oil phase and headspace. Plotted is the discontinuous CO_2_ measurement of three biological replicates (A). The surface tension of free water phases under the tested oil phases after 1 and 2 weeks with and without microbes is shown. Plotted are up to three biological replicates (B). The pH of the free water phases after 1 and 2 weeks with and without microbes is shown. Plotted are up to three biological replicates (C).

To analyze the effect of GtL fuels and HVOs on microbial activity during heating oil storage, HEL3 was chosen as a base fossil heating oil. The fossil heating oil was compared in a 17‐day storage culture (5:1 culture) with a commercial admixture of 50% GtL or 50% HVO, as well as with oil phases of pure GtL and pure HVO. Under the fossil heating oil, microbial activity of about 24 mg CO_2_ was measured. The admixture of 50% HVO resulted in a 28% reduction to 17 mg CO_2_. The admixture of 50% GtL, pure HVO, and pure GtL allowed a microbial activity of only about 14 mg CO_2_, which corresponds to a reduction by 45% (Figure 4A).

In the control samples (water phase without microbes), overlaying with fossil heating oil, with 50% HVO and 50% GtL led to a reduction in surface tension after 1 week (−MO Week 1), which is absent with pure HVO or pure GtL. This reduction in surface tension is compensated by the presence of microbes after 1 and 2 weeks (+MO Week 1 and 2) (Figure 4B). In the water phases of the control samples (water phase without microbes) below pure GtL alone, there was no reduction in pH after 1 week (−MO Week 1). Reductions in pH in the remaining samples were compensated by the presence of microbes after 1 and 2 weeks (+MO Week 1 and 2) (Figure 4C).

**FIGURE 5:**
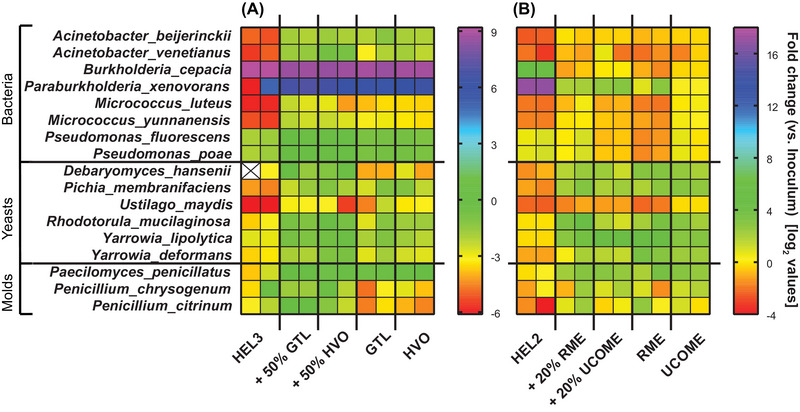
Abundance estimation of microbes by shotgun sequencing following storage cultures. Shown is the result of Illumina sequencing by the Institute of Medical Microbiology and Hygiene, NCCT Microbiology (Tübingen, DE) after cleaning of the Illumina raw data and reads via the “nxf‐bcl” and “nxf‐fastqc” pipeline and alignment against a user‐defined sequence database of the used species by Dr. rer nat. Angel Angelov (Freising, DE). Plotted is the change in reads per species after completion of storage cultures compared to inoculum. A log_2_‐scale was applied to the data. Two biological replicates per oil phase are plotted, and the values are mean values of two technical replicates each. The result for the storage culture series of the GtL/HVO blends is plotted on the left (A) and the result for a storage culture series using RME/UCOME blends as overlay is plotted on the right (B). GtL, gas‐to‐liquid; HVO, hydrogenated vegetable oil.

The change in reads per strain after completion of the 17‐day storage culture (5:1) compared to the inoculum based on shotgun sequencing showed for 50% GtL and 50% HVO compared to fossil heating oil that bacteria and specifically *Burkholderia* sp. continue to dominate the culture, but also that the majority of yeasts and molds can maintain their shares in the culture (exception *U. maydis*). In contrast, sequencing of samples from storage cultures with pure GtL and pure HVO showed a reduction in the proportions of yeasts and molds (*Debaryomyces hansenii*, *Yarrowia deformans*, *Penicillium chrysogenum*, and *Penicillium citrinum*) (Figure 5 middle).

**FIGURE 6:**
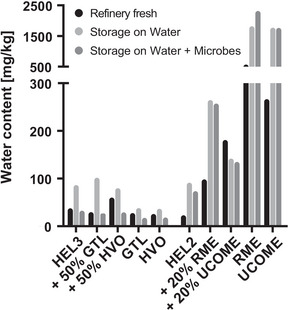
Analysis of the water content in oil phases of 1:5 approaches. The analysis of water content in oil phases of three series of 1:5 approaches is shown. The 1:5 approaches of GtL or HVO blends of the fossil heating oil HEL3 (left) and the 1:5 approaches of RME or UCOME blends of the fossil heating oil HEL2 (right) are shown. Per oil phase, the water content of the refinery‐fresh blend, the water content after 2 weeks of storage on water without and with microbes are plotted. Shown are the mean values of three technical replicates for refinery‐fresh blends or the mean values of technical replicates based on the pooled oil phase of 12 biological replicates when stored on water without and with microbes. GtL, gas‐to‐liquid; HVO, hydrogenated vegetable oil.

**FIGURE 7:**
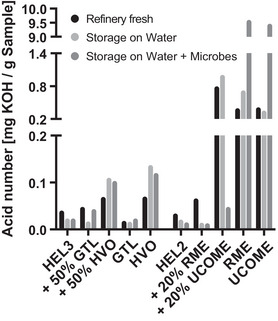
Analysis of the acid number in oil phases of 1:5 approaches. The analysis of acid number in oil phases of two series of 1:5 approaches is shown. The 1:5 approaches of GtL or HVO blends of the fossil heating oil HEL3 (left) and the 1:5 approaches of RME or UCOME blends of the fossil heating oil HEL2 (right) are shown. Per oil phase, the acid number of the refinery‐fresh blend, the acid number after 2 weeks of storage on water without and with microbes are plotted. Shown are the mean values of three technical replicates for refinery‐fresh blends or the mean values of technical replicates based on the pooled oil phase of twelve biological replicates when stored on water without and with microbes. GtL, gas‐to‐liquid; HVO, hydrogenated vegetable oil.

The oil phase analysis indicated reduced water contents of 35 mg/kg to 23–27 mg/kg in the heating oil phases when GtL or HVO were present (50% or 100%). In the case of the pure GtL or HVO oil phases, storage on water for 2 weeks (1:5 culture) resulted in the absence of water enrichment (Figure 6, center). In contrast, the oil phases with an admixture of 50% HVO as well as pure HVO showed an increased acid number after 2 weeks of storage on water and on water with microbes (1:5 culture) (Figure 7 center).

**FIGURE 8:**
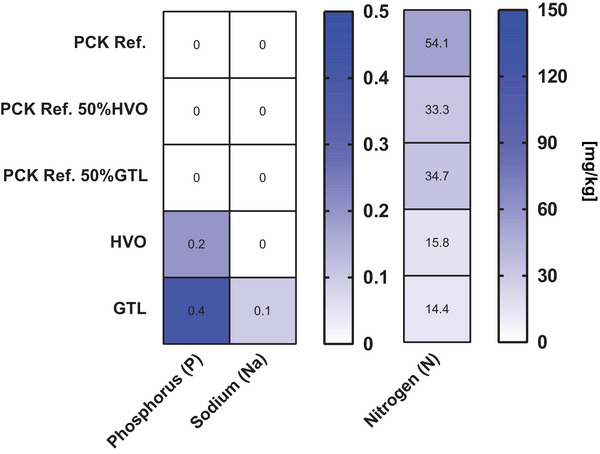
Determination of trace elements and nitrogen contents in alternative paraffinic blends. Mean values of technical replicates are shown. Trace elements below the detection limit of the test are not shown.

The trace element analysis of the oil phases confirmed the contamination of the pure GtL and HVO oil phases with additional phosphorus sources (0.4 and 0.2 mg/kg, respectively). In contrast, the nitrogen analysis showed that the fossil fuel oil HEL3 contains far more nitrogen (54 mg/kg) than the pure GtL or HVO (14 and 16 mg/kg, respectively) (Figure 8).

**FIGURE 9:**
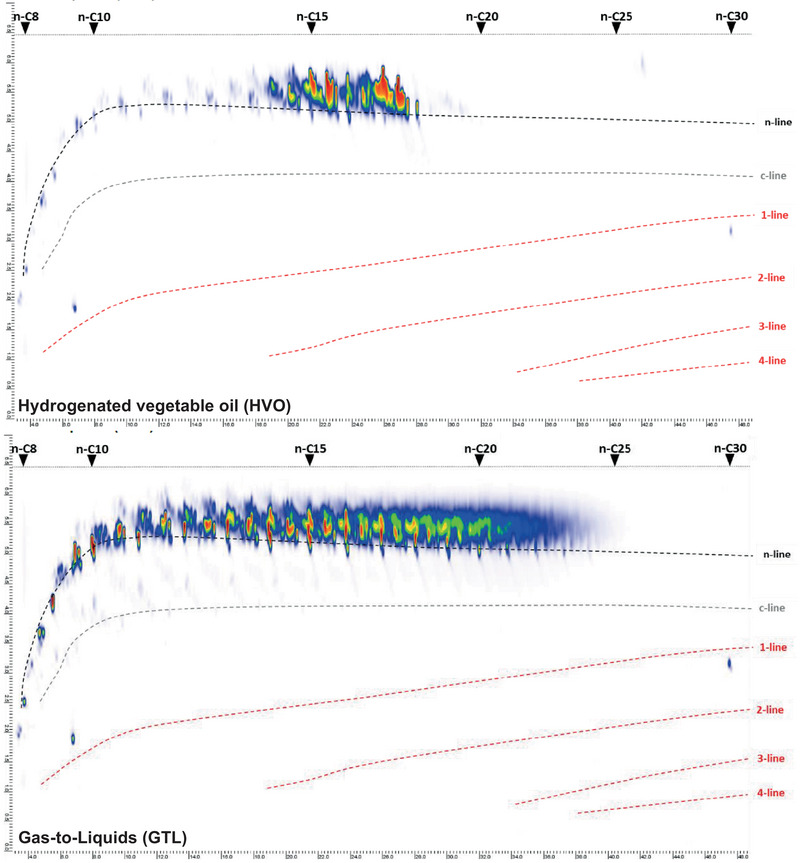
Chemical composition of paraffinic fuels used in this study. A total ion chromatogram of 2D‐GC/MS is shown (Laboratory Lommatzsch & Säger, Cologne). GC, gas‐chromatography; MS, mass spectrometry.

The composition of GtL and HVO was identified by GC × GC/MS and accurately quantified by GC × GC/FID (Table 2 and Figure 9, respectively). It was found that GtL and HVO consist exclusively of n‐/iso alkanes. The alkanes have a chain length range restricted to C11–C20 in the case of HVO, and a broader chain length range of C8–C25 in the case of GtL.

**TABLE 2:** Chemical composition of paraffinic fuels used in this study, results of 2D‐GC/MS (Laboratory Lommatzsch & Säger, Cologne).

HVO	Alkanes		GtL	Alkanes	
(%)	n‐/iso	Cyclo	(%)	n‐/iso	Cyclo
C8–C10	1,2	0,1	C8–C10	7,6	0,3
C11–C15	24,2	0,2	C11–C15	38,3	1,0
C16–C20	73,1	0,6	C16–C20	38,7	0,6
C21–C25	0,2	0,0	C21–C25	13,3	0,2
C26–C30	0,3	0,0	C26–C30	0,1	0,0

GC, gas‐chromatography; GtL, gas‐to‐liquid; HVO, hydrogenated vegetable oil; MS, mass spectrometry.

### 3.3 Microbial activity below future blends of paraffinic fuels and biodiesel

**FIGURE 10:**
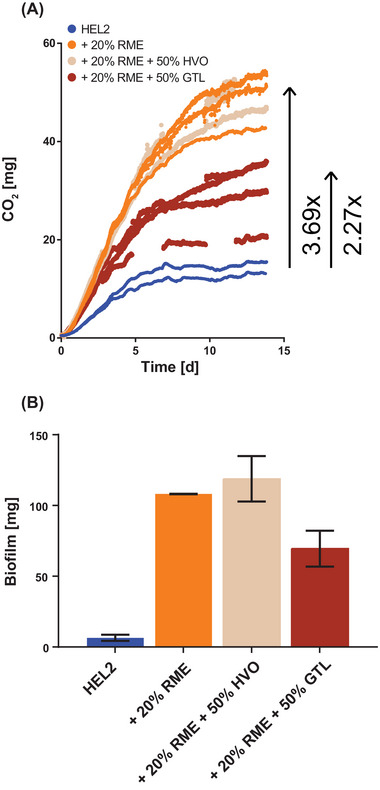
Storage cultures of RME/HVO and RME/GtL blends. Microbial activity in a 14‐day storage culture (5:1) with fossil heating oil HEL2, fossil heating oil with 20% admixture of RME, with 20% admixture of RME plus 50% admixture of HVO, and of 20% admixture of RME plus 50% admixture of GtL is shown. Microbial activity is measured by the sum of CO_2_ accumulation in oil phase and headspace. Plotted is the discontinuous CO_2_ measurement of up to four biological replicates. Plotted are up to four biological replicates (A). The mass of biofilm formed in 14‐day storage cultures (1:5) with fossil heating oil HEL2, fossil heating oil with 20% admixture of RME, with 20% admixture of RME plus 50% admixture of HVO, and of 20% admixture of RME plus 50% admixture of GtL is shown. Mean values and standard deviation of two technical measurements of pooled samples from each of four biological replicates (B) are plotted. GtL, gas‐to‐liquid; HVO, hydrogenated vegetable oil.

To analyze the possible effect of the future combination of alternative paraffinic fuels (GtL or HVO) and biodiesel (RME or UCOME) in heat supply on microbial activity or susceptibility, a 14‐day storage culture series (5:1 culture) was run with 20% RME plus 50% GtL or 50% HVO as oil phase. The comparison was made to cultures with fossil heating oil HEL2 or fossil heating oil with 20% RME. Under fossil heating oil, microbial activity was 15 mg CO_2_ release. Under fossil heating oil with the addition of 20% RME, CO_2_ accumulation of 54 mg was seen, corresponding to a 269% increase in microbial activity. The further addition of 50% HVO did not significantly affect the microbial activity (48 mg CO_2_). In contrast, the addition of 50% GtL allowed only 34 mg CO_2_ microbial activity after 14 days, corresponding to a 127% increase in microbial activity compared to fossil heating oil (Figure 10A).

In addition to monitoring microbial activity, as measured by the accumulation of CO_2_, the possibility of biofilm formation was quantitatively compared depending on the oil phase used. For four pooled biological replicates (1:5 cultures), 6 mg of biofilm was harvested after 2 weeks from the fossil heating oil HEL2. For the addition of 20% RME or 20% RME plus 50% HVO, a 17‐ and 18‐fold increase in measured biofilm to 108 and 119 mg, respectively, was recorded. In contrast, for the addition of 20% RME plus 50% GtL, only an 11‐fold increase to 70 mg biofilm could be documented (Figure 10B).

## 4 DISCUSSION

During the time window of both types of storage cultures (1:5 and 5:1 cultures), the onset of microbial contamination was simulated. This includes selection processes of the microbial mixture used and initial biofilm formation at the interface between the water and oil phase. The production of microbial biosurfactants, emulsion formation, and the increasing spread of microbes into the oil phase in the course of advanced contamination by an adapted microbial mixture was not investigated here.

In addition, a preexisting water phase was used in this work, since the presence of a free water phase is crucial for the progression of microbial contamination (Figure 1) [11, 44].

### 4.1 Toxic degradation products of OME blends prevent microbial activity

The influence of OMEs on microbial activity was investigated by a storage culture series (5:1) with an admixture of 2%–10% OME to fossil heating oil HEL2. The admixture of OME provided complete suppression (85%) of microbial activity in the storage culture (Figure 2A). The parallel lowering of pH and surface tension and the subsequent HPLC‐UV‐RI analysis of control samples without microbes (5:1) (Figure 2B–D) showed that the admixed OME decomposes in contact with the free water phase to its starting products formaldehyde and methanol. Subsequent oxidation of formaldehyde also provided small amounts of formic acid sufficient to lower the pH of the unbuffered water phase. By repeating the storage culture series with buffered water phase (phosphate buffer) and stable pH (data not shown) and by compensating the pH when 2% OME was added to the oil phase by microbes present (<10 mM formic acid, Figure 2C), the pH effect could be ruled out for the inhibition of microbial activity. The long‐term storage cultures with a 10% OME admixture to fossil heating oil HEL2 (Figure 3) and regular measurement of extractable gDNA as a measure of the biomass present also showed that the antimicrobial effect of formaldehyde and methanol persisted for at least 1 year and that no rapid adaptation of the broad microbial mixture took place. Our documentation confirms existing reports on acid‐catalyzed back reactions of OMEs to the starting products methanol and formaldehyde [45], which is determined by the availability of water present [35, 46]. It seems obvious to exploit or commercialize the additional antimicrobial or preservative effect of OMEs or their decomposition products in contact with infiltrated water, as identified here. Given the ongoing registration with the European Chemicals Agency (Cas No. 30846‐29‐8 or 66455‐31‐0) and the ongoing preparation of a standard for the power and fuel sector (DIN/TS 51699), it is important to declare the antimicrobial effect as a side effect and to avoid classification as a biocide under the Biocidal products regulation (BPR). The classification as a side effect is supported by the fact that the antimicrobial effect only comes into play from an addition of 1%–2% v/v to the fuel. The typical use of biocides in the fuel sector is <100 ppm. The existing registration of the released formaldehyde as a biocide (CAS No. 50‐00‐0) and the possible violation of occupational exposure limits for formaldehyde of 0.37 mg/m^3^ when released into the gas phase have to be taken into account for regulatory classification and application, though.

### 4.2 Limited microbial activity below gas‐to‐liquids and hydrogenated vegetable oils

The influence of the alternative paraffinic fuels was investigated using HVO and a GtL, a representative of the Fischer–Tropsch products (XtL). Blending the market standard 50% HVO resulted in a 28% reduction in potential CO_2_ accumulation in comparison while blending 50% GtL and pure HVO and GtL resulted in a 45% reduction to 13–15 mg CO_2_ (Figure 4A). This corresponds to the minimum microbial activity observed for fossil heating oils (Figure S2). One reason, not further discussed here, is for sure the limitation of the diversity of carbon sources to a single challenging one.

In parallel, under oil phases with the paraffinic alternatives, a continuous stabilization of the surface tension of the water phases (Figure 4B) and, associated with this, a reduction of the water content in untreated oil phases as well as an absence of water accumulation in the storage cultures (Figure 6 middle) could be observed. This corresponds to lower availability of surface active and potentially simple nutrients from the oil phase. There is also a lower likelihood of emulsions and microbial dispersal into the oil phase itself, spread within the oil tank, or burner system (https://docplayer.org/76103112‐Herstellung‐thg‐reduzierter‐fluessiger‐kraft‐und‐brennstoffe‐herstellung‐thg‐reduzierter‐fluessiger‐kraft‐und‐brennstoffe‐kurzstudie.html, accessed 20 December 2021). Nevertheless, separate free water phases are expected to form even faster via condensation. The water enrichment could be shown for fossil oil phases following storage on water. Microbes degrade available surface active substances and prevent the enrichment during the onset of microbial contamination (Figure S3). While stabilization of the pH in the water phase was observed for the admixture of GtL (Figure 4C), the admixture of HVO provided slightly increased acid numbers in the oil phase due to storage on water (Figure 7 middle). The hydrolysis‐induced release of fatty acids from present traces of FAMEs in the case of HVO is certainly due to the plant feedstock, but is by no means comparable to the extent in the case of biodiesel blending (Figure 7 right) and did not lead to increased microbial activity for the time being.

The blending of GtL and HVO resulted in an increase in phosphorus content and, although not in more nitrogen overall (14–15 mg/kg for pure GtL and HVO, respectively), in additional simple inorganic nitrogen sources. The low overall nitrogen content of the paraffinic alternatives was unexpected because the provisional technical specification DIN/TS 51603‐8 includes a nitrogen limit of 140 mg/kg, implying a high availability in general (https://docplayer.org/76103112‐Herstellung‐thg‐reduzierter‐fluessiger‐kraft‐und‐brennstoffe‐herstellung‐thg‐reduzierter‐fluessiger‐kraft‐und‐brennstoffe‐kurzstudie.html, accessed 20 December 2021) [29]. The fossil fuel oil HEL3 contained large amounts (54 mg/kg) of polyaromatic nitrogen sources (Figure S4). Thus, the admixture of HVO and GtL gave yeast and mold additional elements for growth C:N:P ratio (Figure 8). The impact of nitrogen availability for microbial activity in storage cultures could be shown by a separate storage approach (Figure S5). Sequencing showed that the 50% admixture of HVO and GtL allowed increased microbial diversity by stabilizing the proportions of yeasts and molds in the presence of bacterial dominance (*Burkholderia* sp.) (Figure 5 middle). In contrast, a renewed restriction of microbial diversity was observed for oil phases of pure GtL and HVO. The crucial difference was the restriction to n‐/iso alkanes as the only carbon source and the omission of aromatics. Thus, in addition to N and P availability, the supply of species‐dependent simple or diverse carbon sources is crucial for microbial diversity.

The 50% admixture of GtL to fossil heating oil HEL3 resulted in a stronger decrease in microbial activity than the 50% admixture of HVO (Figure 4A). Besides the hydrolysis‐induced release of free fatty acids as simple carbon sources in the case of HVO, the GC × GC/MS and GC × GC/FID analysis of the oil phases provided further reasons (Table 2 and Figure 9, respectively). The GtL used possessed a higher proportion of n‐/iso alkanes of shorter chain lengths. Short‐chain alkanes react non‐specifically with the lipid membrane of microorganisms and have toxic effects [47, 48, 49]. To confirm the toxic effect of GtL, an additional storage culture series was run in which the admixture of 50% HVO or 50% GtL to fossil heating oil HEL2 with 20%RME was investigated (Figure 10A). The admixture of 20%RME resulted in an increase in microbial activity from 15 to 54 mg CO_2_. This result was unaffected by the addition of 50% HVO (48 mg CO_2_). But the admixture of 50% GtL resulted in reduction of microbial activity to 34 mg CO_2_ after 2 weeks. In this culture series in the 5:1 approach, however, the subjective impression also arose that the biofilm formation between the water and oil phases was not affected by the toxic effect, but on the contrary, was even stronger with the admixture of 50% HVO or 50% GtL. Since the formation of biofilm poses a crucial threat to the functionality of the burner systems, a third culture series was performed in a 1:5 format to harvest and weigh the biofilms (Figure 10B). Quantification of the biofilms confirmed the CO_2_ monitoring results (microbial activity). Biofilm formation was unchanged by blending 50% HVO to 20% RME (108 and 119 mg, respectively). In contrast, biofilm formation was reduced to 70 mg by blending 50% GtL to 20% RME.

To sum it up, the addition of OMEs revealed an antimicrobial side effect that could be activated by water, the addition of biodiesel confirmed the acceleration of microbial contamination, and the addition of paraffinic fuels, although also partly of biogenic origin, proved to be microbially resistant alternative heating oils. This work shows that fossil heating oils and their CO_2_‐reduced alternatives, whether biogenic or synthetic, impose multiple parameters on the onset of microbial contamination that promote, restrict, complement, or limit the microbial contamination process.

Species‐specific usable nitrogen sources of the oil phases promote microbial activity, while toxic extracts, such as formaldehyde, limit microbial activity. An optimized N and P availability enhances microbial diversity, but is limited by demanding carbon sources.

The use of advanced chromatography, microbial sequencing, and element (nitrogen and phosphorus) analysis can determine in detail the basis of an accelerated or retarded contamination process. Surface tension and pH or acid number and water content at the boundary between infiltrated water and oil phase, as well as CO_2_ accumulation in the storage tank headspace, can be used as indicators of the onset of microbial contaminations.

With this analytical workbench, one can determine the susceptibility of heating oil to microbial contaminations and can use this information for prevention, for example, by additive addition or fuel blending.

## CONFLICT OF INTEREST STATEMENT

The authors declare no conflict of interest.

## Supporting information

Supplementary Figure 1. GC analysis of the OME solution used. Shown is that the mass of all OME components found in the sample is 94.9% of the weighed sample mass. The GC method does not take into account the OME component OME‐6 contained in the sample, which, however, could be observed as a peak and explains the missing mass fraction.

Supplementary Figure 2. Storage cultures of fossil heating oils. Microbial activity in 2‐week storage of fossil heating oils from six German refineries measured by the sum of CO_2_ accumulation in oil phase and headspace is shown. Plotted is the discontinuous CO_2_ measurement of three biological replicates.

Supplementary Figure 3. Influence of microbes on the water content of fossil heating oils during storage cultures. The water content of HEL3 after storage for 2 weeks in 1:5 approaches on 0.1% NaCl plus microbes (A) and on 0.1% NaCl (water) (B) is shown. In addition, the water content of the untreated heating oil and the water content after storage for 2 weeks initially on 0.1% NaCl (water) followed by storage for 2 weeks on 0.1% NaCl plus microbes is shown (B followed by A). Plotted are individual measurements from up to four biological or technical replicates and the corresponding mean value.

Supplementary Figure 4. Chemical composition of fossil heating oils HEL2 and HEL3 used in this study. The total ion chromatogram of GC x GC/MS is shown (Laboratory Lommatzsch & Säger, Cologne).

Supplementary Figure 5. Relevance of nitrogen for microbial activity in storage cultures of fossil heating oils. Microbial activity in a 14‐day storage culture (5:1 culture) of fossil heating oil HEL3 with a water phase of 0.1% NaCl and with a water phase of 0.1% NaCl plus 2.9 g/L NH_4_Cl is shown. Microbial activity is measured by the sum of CO_2_ accumulation in oil phase and headspace. Plotted is the discontinuous CO_2_ measurement of up to three biological replicates.

## Data Availability

The data that support the findings of this study are available from the corresponding author upon reasonable request.
