# Supplementary material for: Evaluating microbial contaminations of alternative heating oils
Source: Eng Life Sci. 2023 May 5;23(6):e2300010. doi: 10.1002/elsc.202300010 (PMC10235886; doi:10.1002/elsc.202300010)
Supplement: Supplementary file 1 — Supplementary Figure 1. GC analysis of the OME solution used. Shown is that the mass of all OME components found in the sample is 94.9% of the weighed sample mass. The GC method does not take into account the OME component OME‐6 contained in the sample, which, however, could be observed as a peak and explains the missing mass fraction. [file ELSC-23-e2300010-s004.pdf]

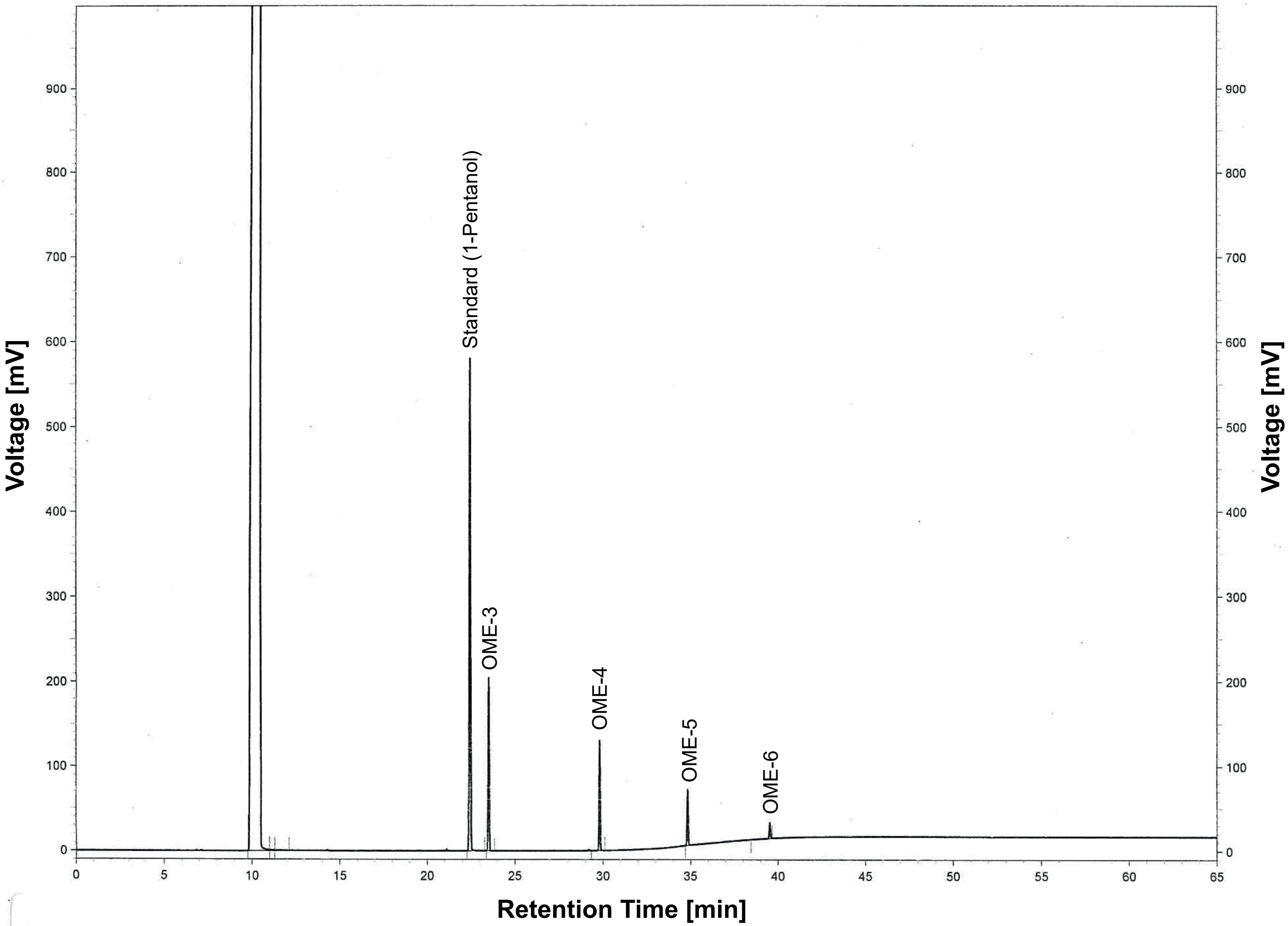

	Correction factor	M [g/mol]	Peak area	Mass [mg]	Mole amount [mmol]	Mass recovery [%]
Total sample				26,90		
Standard (1-Pentanol)	1,00	88,15	26258666	14,60	0,17	
OME-1	2,30	76,10	0	0,00	0,00	
OME-2	3,23	106,06	0	0,00	0,00	
OME-3	3,00	136,15	7648388	12,76	0,09	
OME-4	3,15	166,17	4752387	8,32	0,05	
OME-5	3,15	196,10	2541241	4,45	0,02	
Sum				25,53		94,91408558
