# Supplementary figures and images for: Evaluating microbial contaminations of alternative heating oils
Source: Eng Life Sci. 2023 May 5;23(6):e2300010. doi: 10.1002/elsc.202300010 (PMC10235886; doi:10.1002/elsc.202300010)

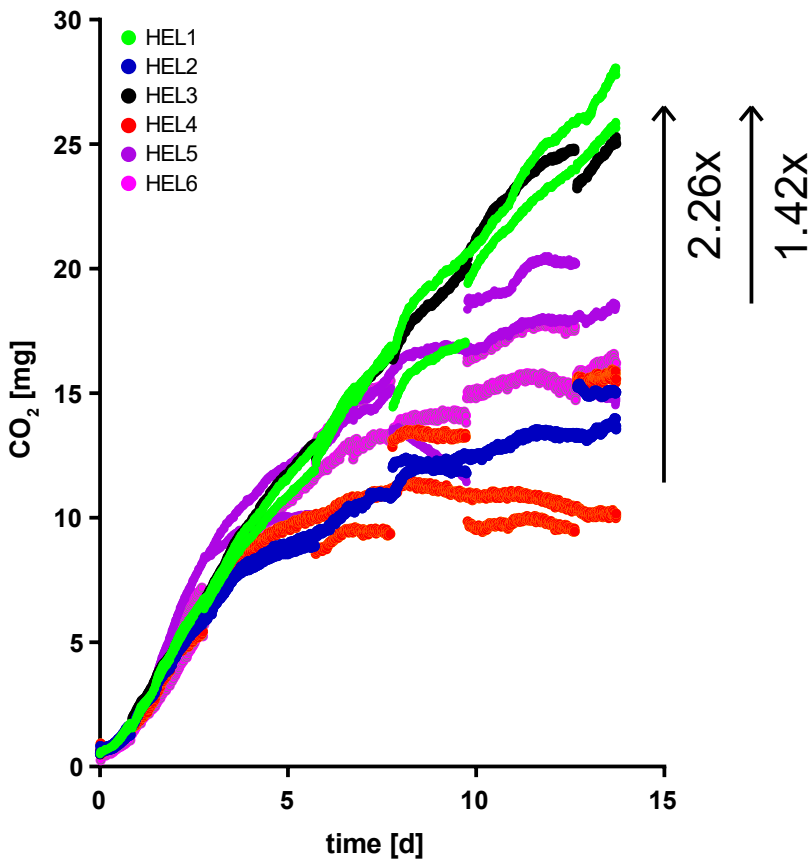

Supplement: Supplementary file 2 — Supplementary Figure 2. Storage cultures of fossil heating oils. Microbial activity in 2‐week storage of fossil heating oils from six German refineries measured by the sum of CO2 accumulation in oil phase and headspace is shown. Plotted is the discontinuous CO2 measurement of three biological replicates. [file ELSC-23-e2300010-s003.pdf]

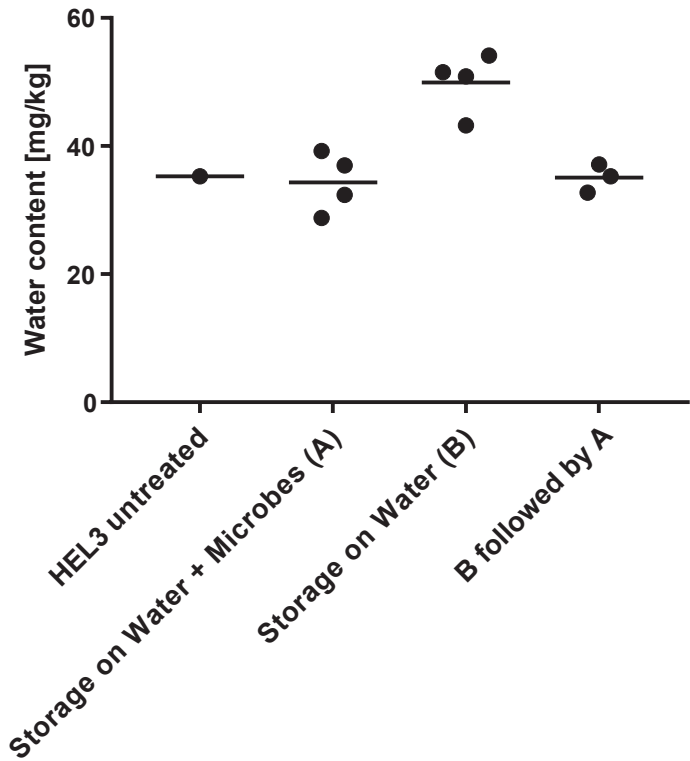

Supplement: Supplementary file 3 — Supplementary Figure 3. Influence of microbes on the water content of fossil heating oils during storage cultures. The water content of HEL3 after storage for 2 weeks in 1:5 approaches on 0.1% NaCl plus microbes (A) and on 0.1% NaCl (water) (B) is shown. In addition, the water content of the untreated heating oil and the water content after storage for 2 weeks initially on 0.1% NaCl (water) followed by storage for 2 weeks on 0.1% NaCl plus microbes is shown (B followed by A). Plotted are individual measurements from up to four biological or technical replicates and the corresponding mean value. [file ELSC-23-e2300010-s001.pdf]

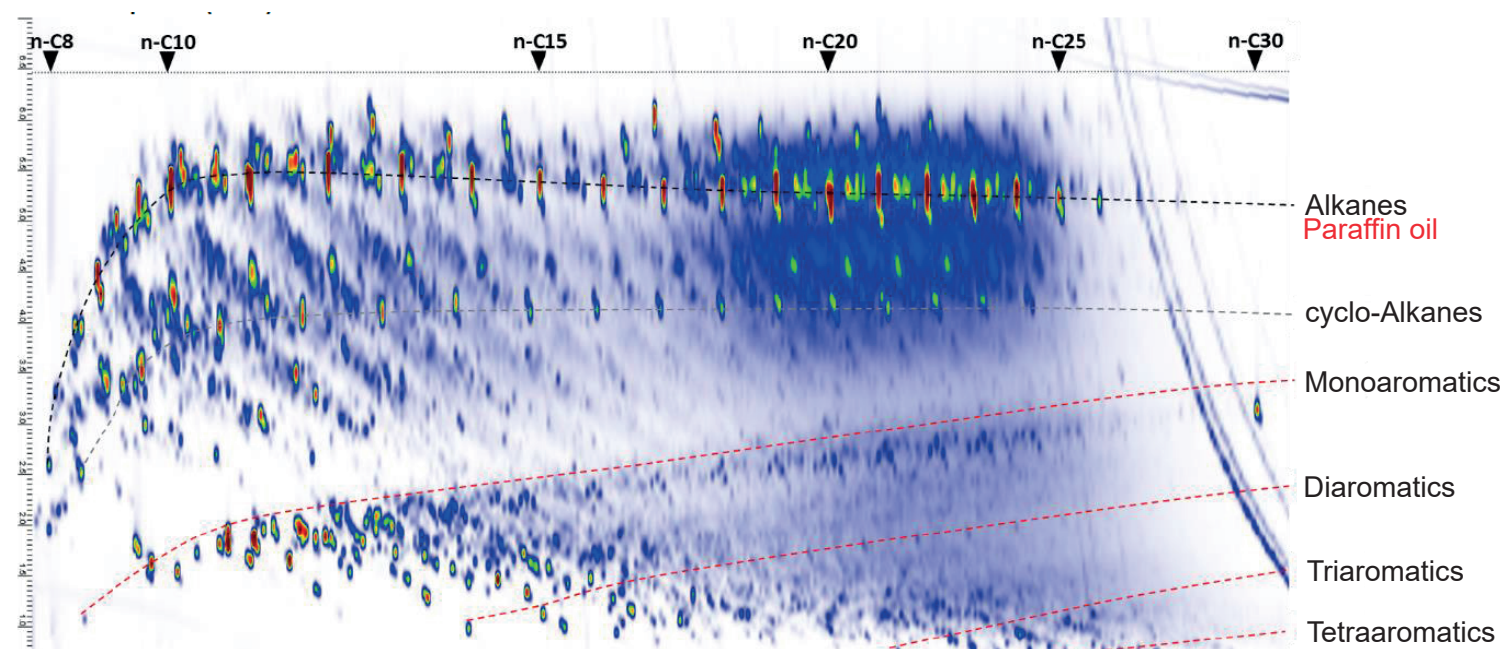

HEL2

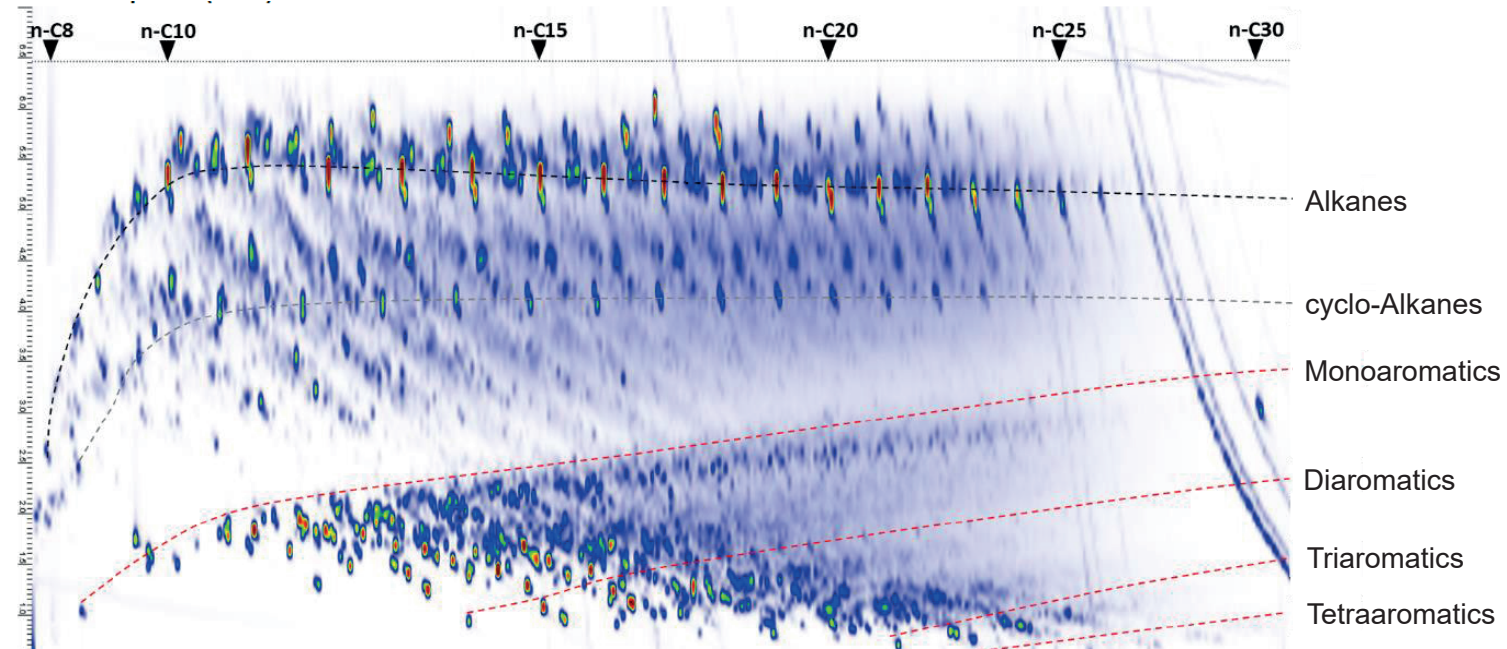

HEL3

Supplement: Supplementary file 4 — Supplementary Figure 4. Chemical composition of fossil heating oils HEL2 and HEL3 used in this study. The total ion chromatogram of GC x GC/MS is shown (Laboratory Lommatzsch & Säger, Cologne). [file ELSC-23-e2300010-s002.pdf]

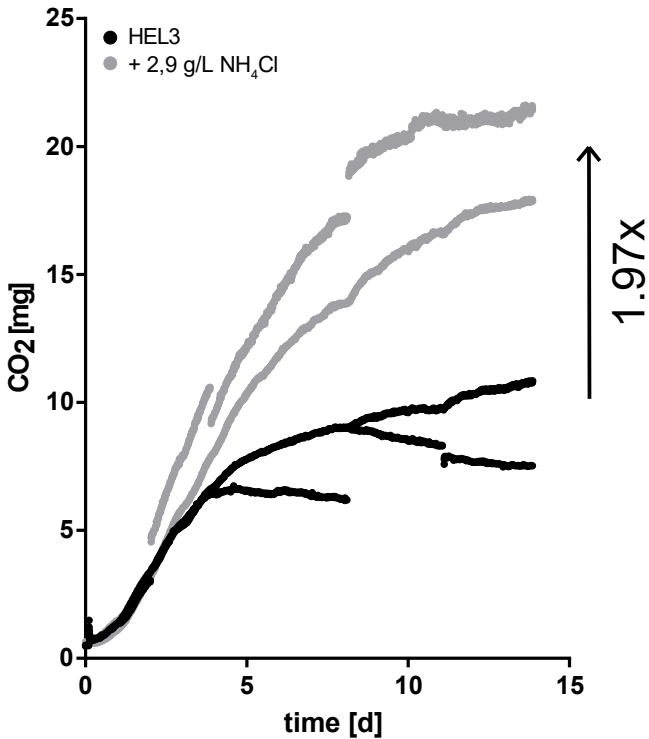

Supplement: Supplementary file 5 — Supplementary Figure 5. Relevance of nitrogen for microbial activity in storage cultures of fossil heating oils. Microbial activity in a 14‐day storage culture (5:1 culture) of fossil heating oil HEL3 with a water phase of 0.1% NaCl and with a water phase of 0.1% NaCl plus 2.9 g/L NH4Cl is shown. Microbial activity is measured by the sum of CO2 accumulation in oil phase and headspace. Plotted is the discontinuous CO2 measurement of up to three biological replicates. [file ELSC-23-e2300010-s005.pdf]
